# Intramolecular *ex vivo* Fluorescence Resonance Energy Transfer (FRET) of Dihydropyridine Receptor (DHPR) β_1a_ Subunit Reveals Conformational Change Induced by RYR1 in Mouse Skeletal Myotubes

**DOI:** 10.1371/journal.pone.0131399

**Published:** 2015-06-26

**Authors:** Dipankar Bhattacharya, Andrew Mehle, Timothy J. Kamp, Ravi C. Balijepalli

**Affiliations:** 1 Cellular and Molecular Arrhythmia Research Program, Department of Medicine, University of Wisconsin-Madison, Wisconsin, United States of America; 2 Department of Medical Microbiology and Immunology, University of Wisconsin-Madison, Wisconsin, United States of America; University of Canberra, AUSTRALIA

## Abstract

The dihydropyridine receptor (DHPR) β_1a_ subunit is essential for skeletal muscle excitation-contraction coupling, but the structural organization of β_1a_ as part of the macromolecular DHPR-ryanodine receptor type I (RyR1) complex is still debatable. We used fluorescence resonance energy transfer (FRET) to probe proximity relationships within the β_1a_ subunit in cultured skeletal myotubes lacking or expressing RyR1. The fluorescein biarsenical reagent FlAsH was used as the FRET acceptor, which exhibits fluorescence upon binding to specific tetracysteine motifs, and enhanced cyan fluorescent protein (CFP) was used as the FRET donor. Ten β_1a_ reporter constructs were generated by inserting the CCPGCC FlAsH binding motif into five positions probing the five domains of β_1a_ with either carboxyl or amino terminal fused CFP. FRET efficiency was largest when CCPGCC was positioned next to CFP, and significant intramolecular FRET was observed for all constructs suggesting that *in situ* the β_1a_ subunit has a relatively compact conformation in which the carboxyl and amino termini are not extended. Comparison of the FRET efficiency in wild type to that in dyspedic (lacking RyR1) myotubes revealed that in only one construct (H458 CCPGCC β_1a_ -CFP) FRET efficiency was specifically altered by the presence of RyR1. The present study reveals that the C-terminal of the β_1a_ subunit changes conformation in the presence of RyR1 consistent with an interaction between the C-terminal of β_1a_ and RyR1 in resting myotubes.

## Introduction

Contraction of a skeletal muscle cell is initiated by a depolarization, which in turn triggers an increase in cytosolic Ca^2+^. The process that transduces membrane depolarization into a cytosolic Ca^2+^ increase is controlled by two highly specialized ion channel complexes, namely the dihydropyridine receptor (DHPR) L-type Ca^2+^ channel in the sarcolemma and the ryanodine receptor type 1 (RyR1) present in the sarcoplasmic reticulum. In skeletal muscle, protein-protein interactions between the pore subunit of the DHPR (œ_1S_) and RyR1 are extensive, [1–9]. The α_1S_ subunit has the familiar topology of a four-repeat voltage-gated channel in which long loops, of ~50 to 150 residues, connect the internal repeats [10]. 3-D reconstructions of skeletal DHPR particles and models of DHPR tetrads suggest that the cytosolic loops of the pore subunit are in intimate contact with the foot structure of RyR1 [11, 12]. The protein-protein interactions between the cytosolic ß_1a_ subunit of the DHPR and RyR1 have also been established [13, 14]; however, the precise role of DHPR ß_1a_ in excitation-contraction coupling is not well defined.

Multiple functional roles of DHPR ß subunits have been identified. DHPR ß subunits strongly modulate the gating of Ca^2+^ channels and also promote the trafficking of channels to the surface membrane [15–22]. Four different genes encode DHPR ß subunits in mammals with multiple splice variants identified for each gene. Knockout of the mouse ß_1_ gene using gene targeting results in the complete absence of excitation-contraction (E-C) coupling in skeletal muscle, which can be rescued by heterologous expression of ß_1_ in knockout myotubes [23]. The impact of ß_1_ knockout in the mouse model appears to be primarily due to a lack of membrane trafficking of œ_1S_ to the sarcolemma [24]. Expression studies in ß_1_ KO myotubes have demonstrated that a wide range of different ß subunit isoforms promote membrane trafficking of œ_1S_ rescuing L-type Ca^2+^ currents, but only ß_1a_ and ß_1c_ can specifically rescue skeletal muscle type excitation-contraction coupling [25–28]. Thus the ß_1_ subunit plays a highly specific role in skeletal muscle E-C coupling in addition to its role in promoting membrane trafficking and modulating gating of the DHPR channel complex.

The voltage-dependent Ca^2+^ channel auxiliary ß subunits are composed of 5 domains including a variable N-terminus, a conserved SH3 domain, a variable linker domain, a conserved GK domain and a variable C-terminus. The core structures of several ß subunits have been solved using X-Ray crystallography and exhibit features representative of the membrane-associated guanylate kinase (MAGUK) superfamily of proteins [29–31]. However, the structures of the N- and C-termini of ß subunits are unknown and how these regions relate to the core of the ß subunit has not been determined. Furthermore, information regarding the conformations and relationship of the ß subunits domains in living cells is not available.

Measurement of fluorescence resonance energy transfer (FRET) efficiency between a donor and an acceptor can provide information on proximity relationships between structural domains of proteins [32]. In this study, we have used an *ex vivo* FRET assay to determine proximity relationships between domains of the ß_1a_ subunit as a means to examine the domain organization of ß_1a_ in living skeletal muscle cells. We have chosen the biarsenical fluorescein derivative FlAsH which exhibits fluorescence upon binding to specific tetracysteine motifs [33–35] as a FRET acceptor and enhanced cyan fluorescent protein (CFP) as a FRET donor [33–37]. Our previous studies and those of others have demonstrated efficient CFP/FlASH tetracysteine FRET in living cells [35] [36] [37]. Tagging ß subunits with fluorescent proteins such as CFP, at least at the N- or C-terminus, does not affect function [38]. On the other hand the advantage of using FlAsH is that the tetracysteine domain required for FlAsH binding is very small and less intrusive than other acceptors of choice such as YFP, and so the inserted tetracysteine motif is much less likely to perturb intrinsic biological function of the protein [35, 36]. Furthermore, i) FlAsH binds to proteins tagged with the CCPGCC motif with picomolar affinity; ii) the reagent is cell-permeant; iii) bound FlAsH can be effectively displaced with 1,2-ethanedithiol (EDT); and iv) unbound FlAsH is, for all practical purposes, non-fluorescent [34]. Thus, a FlAsH labeling protocol in cultured cells is easy to implement, and FRET efficiency can be directly quantified from the quenching of CFP emission in the presence and absence of FlAsH [39]. However, FlAsH suffers from two potential limitations: accessibility of FlAsH to the tetracysteine site and background labeling of myotubes [40] that, in principle, could compromise the measurement of the FRET efficiency. In the present work, we have directly addressed both issues. We detected robust intramolecular FRET at 10 donor/acceptor positions within the DHPR ß_1a_ subunit and identified a single donor/acceptor position in which FRET efficiency specifically changed in response to RyR1 expression.

## Materials and Methods

### Ethical approval

The procedure for mouse skeletal myotube isolation was fully approved by the Institutional Animal Care and Use Committee of the University of Wisconsin School of Medicine and Public Health.

#### Mouse models

Heterozygous mice RyR1^+/-^ were a gift of Dr. Hiroshi Takeshima of International Institute for Advanced Studies, Japan [41]. Outbred C57BL/6 mice (Charles River, MA) were used to generate colonies of heterozygous dyspedic (RyR1^+/-^) mice. RyR1 null (RyR1^-/-^) mice were generated by interbreeding of heterozygous parents. PCR primers 5’ GGA CTG GCA AGA GGA CCG GAG 3’ and 5’ GGA AGC CAG GGC TGC AGG TGA GC 3’ were used to amplify a 400-bp fragment of the WT RyR1 allele. PCR primers 5’ GGA CTG GCA AGA GGA CCG GAG 3’ and 5’ CCT GAA CGA GAT CAG CCT CTG TTC C 3’ were used to amplify a 300-bp fragment of the KO RyR1 allele. The PCR cycles for both RyR1 alleles are as follows: initial denature at 94°C for 5 min followed by 35 cycles of 1 min at 94°C, 1 min at 57°C and 1 min at 72°C and finally one cycle for 10 min at 72°C.

Heterozygous DHPR ß_1_^+/-^ mice were generated at the transgenic mouse core facility at the University of Wisconsin-Madison [23]. ß_1_ null (ß_1_^-/-^) null mice were obtained by interbreeding of heterozygous parents. PCR primers 5’ GAG AGA CAT GAC AGA CTC AGC TCG GAG A 3’ and 5’ ACA CCC CCT GCC AGT GGT AAG AGC 3’ were used to identify a 250 bp fragment of WT ß1 allele. PCR primers 5`ACA CCC CCT GCC AGT GGT AAG AGC 3`and 5`ACA ATA GCA GGC ATG CTG GGG ATG 3’ to amplify a 197 bp fragment for identifying the KO ß_1_ allele. The PCR cycles for both DHPR ß_1_ alleles are as follows: initial denature at 94°C for 2 min followed by 30 cycles of 30 sec at 94°C, 45 sec at 60°C and 1 min at 72°C and finally one cycle for 10 min at 72°C.

#### CFP/tetracysteine cDNAs

For calibration of *ex vivo* FRET measurements, we used a CFP-tetracysteine concatemer. The 17 amino acid long FlAsH binding motif AEAAAREACCPGCCARA (here on referred as CCPGCC) as previously described [42] was introduced in frame in the C-terminus of ECFP by PCR techniques. The PCR product was directly cloned into ECFP-C1 vector (Clontech, CA)(construct 1). For determination of relative proximity within the DHPR ß_1a_ subunit, we inserted the FlAsH binding motif at five positions in the ß_1a_ sequence and fused CFP to either the N-terminus or C-terminus of ß_1a_ (constructs 8, 12 and 14 to 21). Full-length ß_1a_ (1–524) (GenBank accession No. NM_031173) was cloned in frame to both ECFP-N1 and ECFP-C1 vectors (Clontech, CA) for fusion of CFP to the N- or C-termini of ß_1a_. Insertion of the above described 17-amino acid FlAsH binding motif in different positions was done by two-step PCR strategies. Six to nine base primers were designed to match cloning sites, and to introduce the FlAsH-binding motif. PCR products were cloned into ECFP-N1- ß_1a_ (constructs 8, and 14 to 17) and ECFP-C1- ß_1a_ (constructs 12 and 18 to 21) vectors at the following sites: *EcoRI/MluI* sites for inserting the 17-mer immediately before ß_1a_ M1, after ß_1a_ Q57, and after ß_1a_ P249; *MluI/SalI* sites for inserting the 17-mer after ß_1a_ H458 and after ß_1a_ M524. The resulting products were confirmed by sequencing. To confirm the specificity of FlAsH labeling in the myotubes system, we generated and tested five different CFP-teracystine concatemer constructs (constructs 1–4,) and a CFP construct without any tetracysteine motif (construct 5). To determine the FlAsH accessibility we generated and tested five additional constructs with the insertion of CFP-CCPGCC motif in five different domains (as mentioned above) of full-length ß_1a_ (constructs 8 to 12). We also made two individual constructs either CFP fused (construct 6) or CCPGCC fused (construct 7) in the N-terminal of β_1a_.

#### Primary myotube cultures

Primary cultures of myotubes were prepared from enzyme-digested mouse hind limbs of wild type, RyR1 KO (dyspedic) and ß_1_ KO embryos (E18) as described earlier [43],[44]. Briefly, the muscles were dissected from fetuses and treated with 0.25% (w/v) trypsin, 0.05% (w/v) pancreatin and 0.1% (v/v) penicillin-streptomycin followed by centrifugation and collection of the cells. Mononucleated cells were resuspended in DMEM containing low glucose, 10% horse serum, 10% FBS, 2% chicken serum extract and 0.1% (v/v) penicillin-streptomycin. After plating the cells (~1X10^4^ cells/dish) on 1% gelatin coated 35 mm plastic dishes, the cultures were grown at 37°C in 8% CO_2_ incubator for five to six days. At the myoblast fusion stage, the medium was replaced with DMEM, 10% horse serum, 1.25% chicken embryo extract and 0.1% penicillin-streptomycin, and CO_2_ was decreased to 5%. All the transfections were performed at the myoblast fusion stage with TransIT-LT1 (Mirus Corporation, WI). Cells were incubated with transfection solution containing LT1 and cDNA of interest (5:1 μg ratio) for 3 hours. In case of RyR1 transfected dyspedic myotubes, a separate expression vector encoding the T cell membrane antigen CD8 were mixed, as a transfection marker, and triply transfected [H485 CCPGCC ß_1a_ CFP (construct 21), RyR1 and CD8 constructs] with the LT1. *Ex vivo* FRET and whole-cell analyses of Ca^2+^ currents were performed after 72 hours following transfection. Triply transfected cells were recognized by incubation with CD8 antibody-coated beads (Dynal, Norway) and the transfection efficiency was ~80%.

#### Electrophysiology

Whole-cell voltage-clamp electrophysiology was performed on myotubes using Axopatch 200B amplifier (Axon Instruments, Foster City, CA) with pClamp 7, software. The patch pipettes were pulled from thin walled borosilicate glass capillaries (World Precision Instruments, Inc., Sarasota FL) on Sutter P-87 micropipette puller (Sutter Instrument Co) and polished using microforge MF900 (Narishige). All the experiments were carried out at room temperature with pipette resistance of 1.5–2.5MΩ [44]. The external solution was (in mM) 130 tetraethylammonium-methanesulfonate, 10 CaCl_2_, 1 MgCl_2_, 10 Hepes-tetraethylammonium (OH), pH 7.4. The pipette solution was (in mM) 140 Cs-aspartate, 5 MgCl_2_, 5 EGTA, 10 4-morpholinepropanesulfonic acid-CsOH (pH 7.2).

#### FlAsH labeling and myotube fluorescence

FlAsH labeling was performed with modifications to the protocols described previously [34, 36]. The culture plates were thoroughly rinsed with a rodent Ringer’s solution (in mM: 146 NaCl, 5 KCl, 2 CaCl_2_, 1 MgCl_2_, 10 HEPES, pH 7.4) supplemented with 10 mM glucose and 1 mM Na-pyruvate. The cells were equilibrated for 30 minutes in the dark by adding 2 ml of rodent Ringer’s solution in plates. Each plate was covered with 250 μl of a freshly-made loading solution consisting of 10 μM EDT (Aldrich, WI) and 1 μM FlAsH-EDT_2_ (named Lumio Green from Invitrogen, CA) in rodent Ringer’s solution and incubated at room temperature for 2 hours in the dark with gentle shaking. FlAsH loaded myotubes were thoroughly washed with rodent Ringer’s solution before imaging. Myotube epifluorescence was captured with a cooled CCD DP-70 camera (Olympus) using a 20X/0.95 NA water-immersion objective in an upright Olympus BX51 water-immersion microscope. The sample was excited using 100W Hg lamp and light filtered with standard band-pass excitation/emission filter cubes (ex436/20 nm, em480/30 nm, dichroic455 for CFP, and ex500/20 nm, em535/30 nm, dichroic515 for fluorescein). Image acquisitions of emission of both CFP and FlAsH before and after clearance of FlAsH with EDT were made as described earlier [36]. In brief, one cell per dish was selected and the position of the dish was fixed on microscope stage once the region of interest was selected and imaged. FlAsH removal was accomplished by superfusion with EDT (7.5 mM final concentration) and 2% DMSO in same rodent Ringer’s solution. Cells were washed carefully under direct microscopic visualization, and only cells that showed no detectable change in localization were analyzed. This allowed imaging from same area of the cells before and after FlAsH clearance. Within ten minutes after adding EDT, fluorescein intensity in the myotubes was below detectable levels, and the cell was imaged again. Image analysis was performed using the ImageJ software (Rasband, W.S., ImageJ, U. S. National Institutes of Health, Bethesda, Maryland, USA, http://imagej.nih.gov/ij/, 1997–2014) and [45]. The FRET measurements and analysis were accomplished according to a previously described method by us [36]. A scale of 0 to 225 was used for analyzing of mean pixel intensities of the cells by selecting low to mid expressions. The FRET ratio was calculated by CFP emission after FlAsH loading and after EDT)/(CFP emission after FlAsH loading and before EDT followed by calculation of FRET efficiency = 1-[1/(FRET ratio)] [39]. The FRET efficiency is directly related to the distance between CFP and FlAsH fluorophores according to Förster theory [46].

#### Structural model

The ß_1a_ subunit structure was predicted with Phyre2 [47]. The highest confidence model was obtained using ß_3_ as a template (PDB 1VYU) [29, 47]. The model includes residues 72–175 in the SH3 domain and residues 265-322/383-458 in the GK domain.

#### Statistics

Data are presented as means ± standard error of the mean. Comparisons between two groups were done using student’s t-test. For multiple comparisons, ANOVA was performed with subsequent Bonferroni t-test comparing test groups to the control group. Statistical significance was defined at a value of p<0.05.

## Results

### FlAsH-mediated ex vivo FRET in myotubes

**Fig 1:**
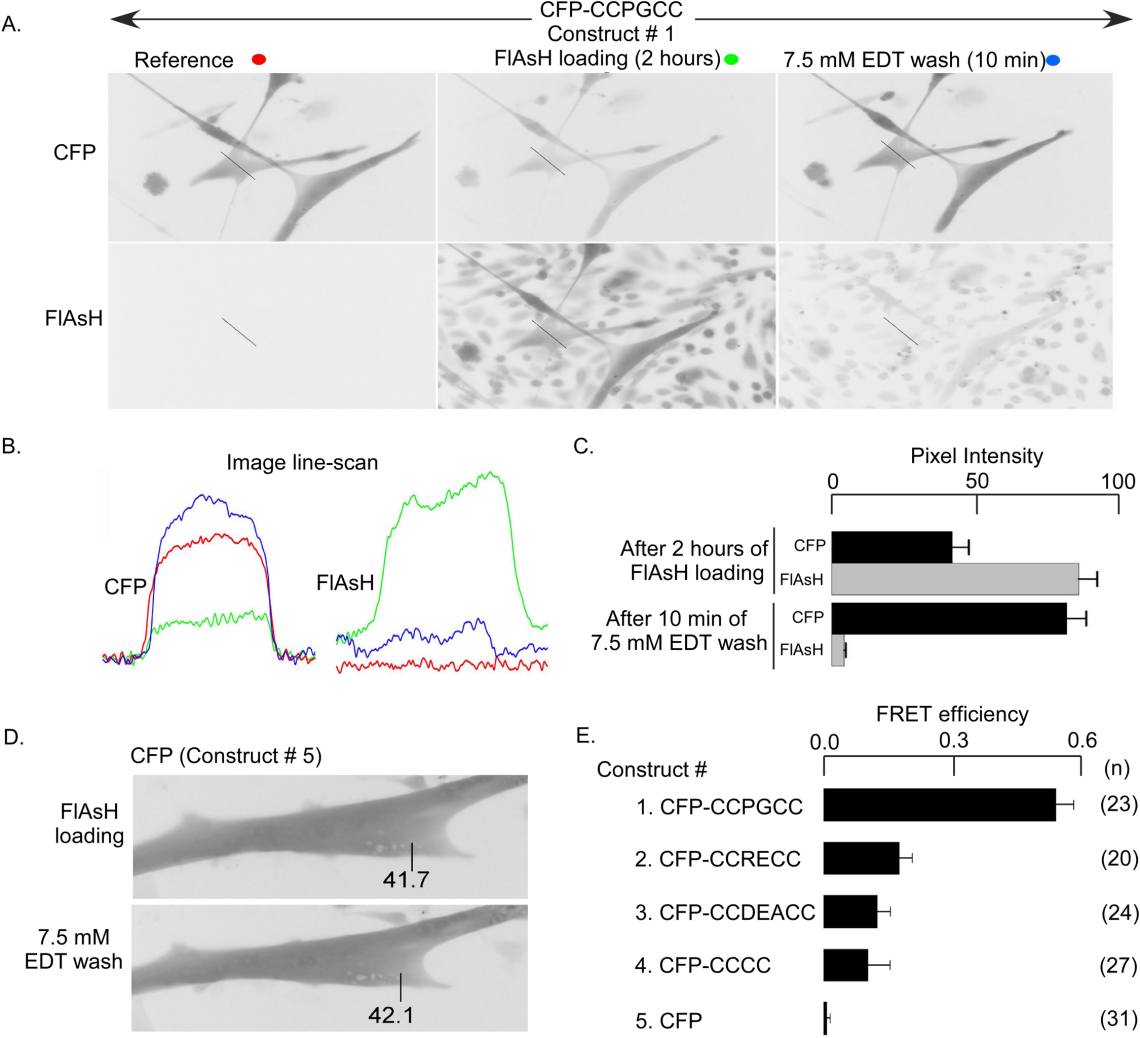
FRET signals in myotubes expressing CFP-CCXXCC and WT CFP. (A) CFP-CCPGCC concatamer (construct 1) transfected myotubes show a high level of CFP expression as indicated by fluorescence in the CFP channel. Before FlAsH loading there was no fluorescence in the FLUO channel for the reference condition (red circle, left panels). After 2 hrs of incubation in 1 μM FlAsH to load the tetracysteine motif, fluorescence in the CFP channel decreased and in the FLUO channel increased (green circle, middle panel). Incubation with 7.5 mM EDT for 10 minutes to displace FlAsH from the tetracysteine tag reversed the fluorescence changes back toward the reference conditions (blue circle, right panels). (B) Pixel intensity of a line scan across the myotube (straight line indicated in each panel of A) in the CFP versus FLUO channels. Colors of traces correspond to color markings in A. (C) Summary of mean pixel intensities of CFP-CCPGCC concatamer (construct 1) expressed in myotubes. (D) WT-CFP (construct 5) transfected myotubes (without the tetracysteine tag) show no change in CFP fluorescence after 1 μM FlAsH loading or after 7.5 mM EDT wash. The number represents the fluorescence intensity (CFP) of a particular region in the myotube. (E) FRET vs FlAsH binding sequences showing CFP-CCPGCC (construct 1) FlAsH complex. Highest FRET efficiency (0.52 ± 0.04) was observed with CFP-CCPGCC (construct 1) than CFP-CCRECC (construct 2, 0.17±0.03), CFP-CCDAECC (construct 3, 0.12 ± 0.03), CFP-CCCC (construct 4, 0.10 ± 0.05) or untagged CFP-CFP (construct 5, 0.07 ± 0.01). Total number of cells (n) analyzed is shown within brackets.

For validation of the FlAsH-based ex vivo FRET in skeletal myotubes, we made several fusion constructs of CFP and the tetracysteine motifs designed to bind FlAsH [36, 42]. The CFP-CCXXCC concatemers were expressed in normal myotubes for 72 hours followed by 2 hours of labeling with 1 μM FlAsH-EDT_2_ in rodent Ringer’s solution. Following FlAsH labeling, cells were incubated with 7.5 mM EDT to track the increase in CFP emission (decrease in FRET) due to the removal of bound FlAsH. Fig 1A shows the changes in donor and acceptor fluorescence in myotubes expressing the CFP-CCPGCC concatemer (construct 1) during the FlAsH labeling protocol. CFP emission decreases after incubation with FlAsH (Fig 1A middle panel), and recovers following FlAsH removal with 7.5 mM EDT (Fig 1A right panel). FlAsH emission is drastically reduced after EDT treatment. The line-scans in Fig 1B show pixel intensity across the width of a cell in the conditions described above in the donor and acceptor channels. To ensure reproducibility and to conform to the definition of FRET efficiency [39], we only focused on cells in which FlAsH removal was substantial (less than 10% residual FlAsH emission after EDT incubation). In the cells with substantial FlAsH unloading by EDT, the CFP emission increased ~2 fold (Fig 1C). The increase in donor emission with concomitant decrease in acceptor emission is a telltale sign of FRET [39]. A robust FRET process, with an efficiency of 0.52 ± 0.04 was measured in the CFP-CCPGCC (construct 1) concatemer expressed in myotubes.

To confirm that the ex vivo FRET signal involved the engineered tetracysteine and not spurious FlAsH acceptors at non-specific sites, we tested CCXXCC tags with different affinities for FlAsH [36, 42], as well as CFP without the tetracysteine tag (Fig 1E construct 1 to 5). The measured FRET efficiency correlated well with the reported affinities of FlAsH for tetracysteine binding sites, which are 4 pM for CCPGCC, 70 pM for CCRECC, 1,800 pM for CCCC and 92,000 pM for CCDEACC [42]. Furthermore, FRET was undetectable in an untagged CFP (Fig 1D and 1E, construct 5). The latter result is important since it confirmed that CFP bleaching during image acquisition and incubation with EDT were insignificant. Thus, the FRET signal only involves the donor and FlAsH acceptor bound to the tetracysteine motif.

### Intramolecular FRET of DHPR ß_1a_ subunit in myotubes

**Fig 2:**
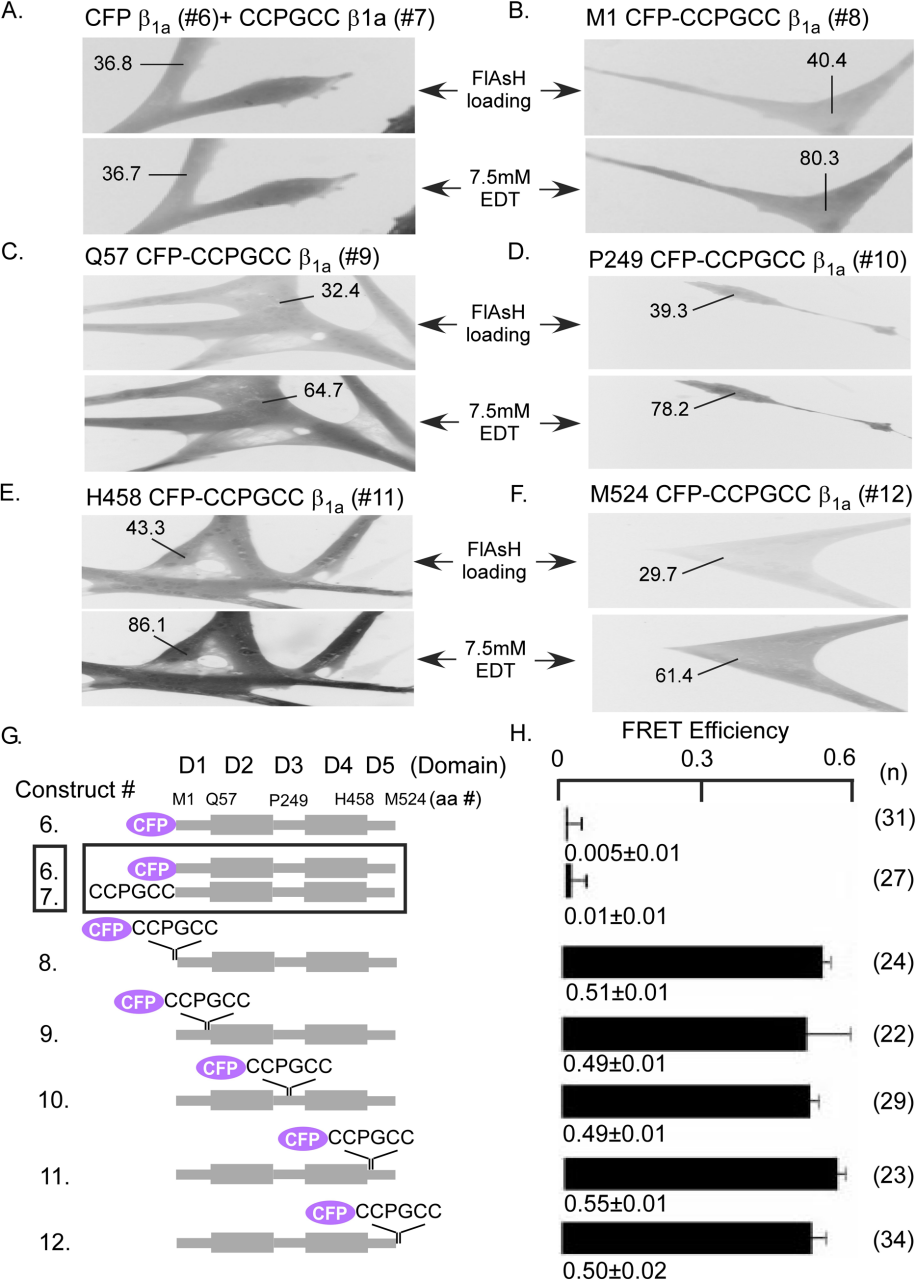
FRET signals from ß_1a_ subunits incorporating the CFP-CCPGCC concatamer in different domains or doubly transfected CFP and CCPGCC tags expressed in WT myotubes. (A) Myotubes co-transfected with CFP- ß_**1a**_ (construct 6) and CCPGCC- ß_**1a**_ (construct 7), constructs demonstrate unchanged FRET efficiency after FlAsH clearance with 7.5 mM EDT indicating that ß_**1a**_ intermolecular interactions do not contribute to FRET in the present study. Myotubes individually transfected with the CFP-CCPGCC concatemers (B) M1 CFP-CCPGCC ß_**1a**_ (construct 8), (C) Q57 CFP-CCPGCC ß_**1a**_ (construct 9), (D) P249 CFP-CCPGCC ß_**1a**_ (construct 10), (E) H458 CFP-CCPGCC ß_**1a**_ (construct 11) or (F) M524 CFP-CCPGCC ß_**1a**_ (construct 12) show ~2 fold FRET efficiency after FlAsH clearance with 7.5mM EDT. The numbers indicated represents the fluorescence intensity of the region in the myotubes before and after FlAsH clearance with EDT for FRET measurements. (G) Schematic diagrams of tagged ß_**1a**_ constructs (constructs 6 to 12) that were used to assess the accessibility of FlAsH to tertracycteine tags incorporated in different domain of ß_**1a**_. (H) Comparison of the FRET efficiencies for different ß_**1a**_ constructs (constructs 6 to 12) expressed in WT myotubes that shows in panel A to G with number of tested cells for each group in parentheses. Mean FRET efficiencies ± standard errors are shown.

After validating the FlAsH-based *ex vivo* FRET system using the CFP-CCPGCC concatemers in myotubes, we sought to evaluate FRET in tagged ß_1a_ subunits. Control experiments revealed that FRET was not detected in the CFP- ß_1a_ fusion that lacked the tetracysteine tag (construct 6) (Fig 2G and 2H), demonstrating that nonspecific binding of FlAsH does not yield any spurious FRET. We also tested for intermolecular FRET between ß_1a_ subunits, given that ß subunits can exhibit intermolecular interaction under certain conditions [48–50]. Co-expressing N-terminal CFP- ß_1a_ and CCPGCC- ß_1a_ in wild type myotubes (Fig 2A) did not result in detectable FlAsH-mediated FRET (Fig 2G and 2H; co-transfected with constructs 6 and 7). Therefore, expression of constructs containing both FRET donor and acceptor in the same ß_1a_ molecule will report intramolecular proximity relationships without the complication of intermolecular FRET between different ß_1a_ subunits.

**Fig 3:**
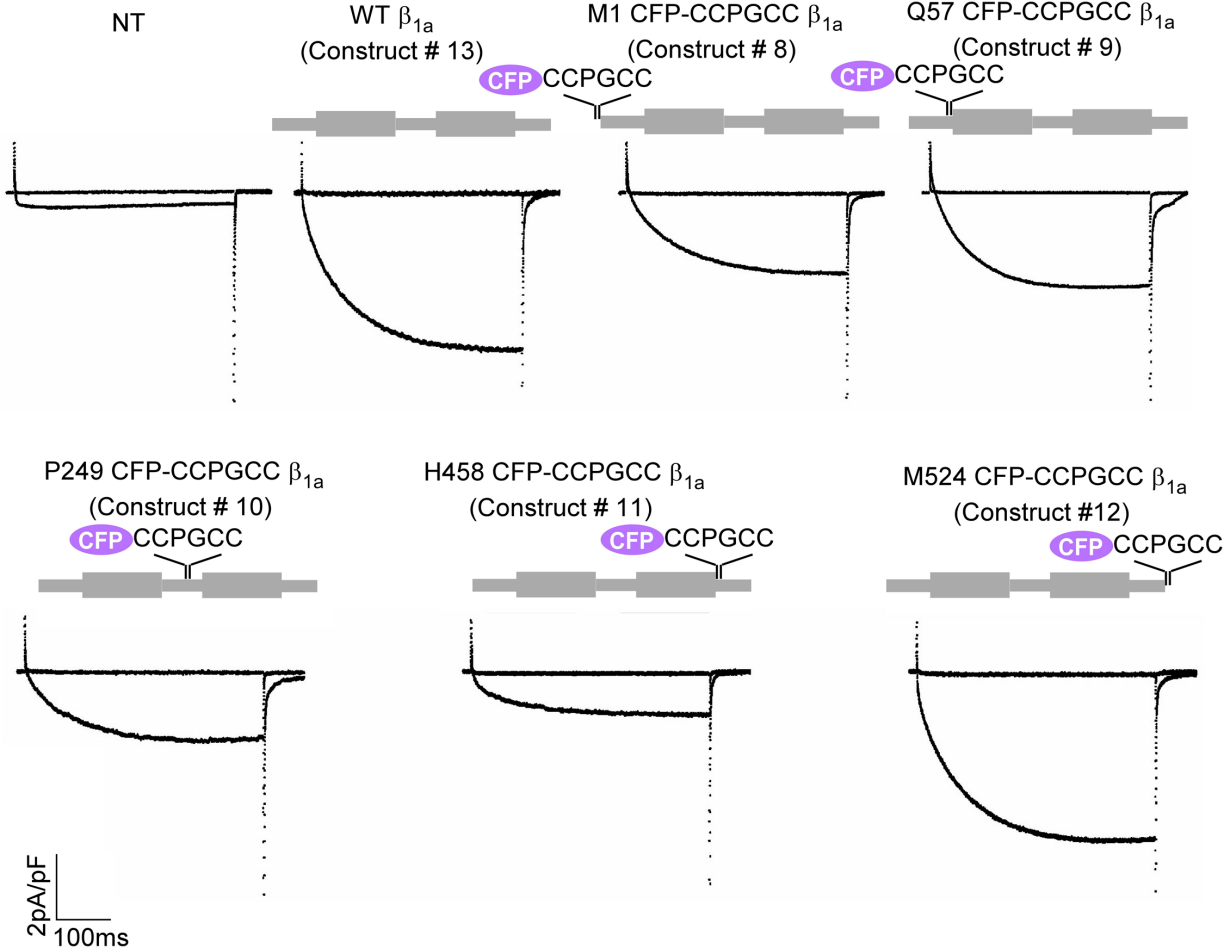
Representative Ca^2+^ current traces resulting from 200 ms test depolarizations to –30 mV and +30 mV in ß_1_ KO myotubes expressing wild-type ß_1a_ (WT ß_1a_ construct 13) and the ß_1a_ CFP-CCPGCC concatemer constructs (constructs 8 to 13) used in Fig 2B–2F or non-transfected (NT) controls.

To determine if accessibility of FlAsH to the CCPGCC motif is a limitation in expressed ß_1a_ subunits, we inserted the CFP-CCPGCC concatemer in five different positions (Fig 2 constructs 8 to 12): the M1 (N-terminal), Q57 (junction of N-terminus and SH3 domains), P249 (the linker between the SH3 and GK domains), or H458 (junction between GK domain and C-terminus;) and M524 (C-terminal). These constructs were expressed independently in wild type myotubes (Fig 2B–2F). FRET efficiency at these five positions in wild type myotubes was similar (Fig 2H constructs 8 to 12) to that of the free concatemer (Fig 1E, construct 1). Thus, the tertiary structure of ß_1a_ does not hinder access of FlAsH to the tetracysteine site. Furthermore, all of these five ß_1a_ CFP-CCPGCC constructs result in large inward I_Ca,L_ when expressed in ß KO myotubes (Fig 3 constructs 8 to 12) which demonstrates the presence of functional ß subunits.

**Fig 4:**
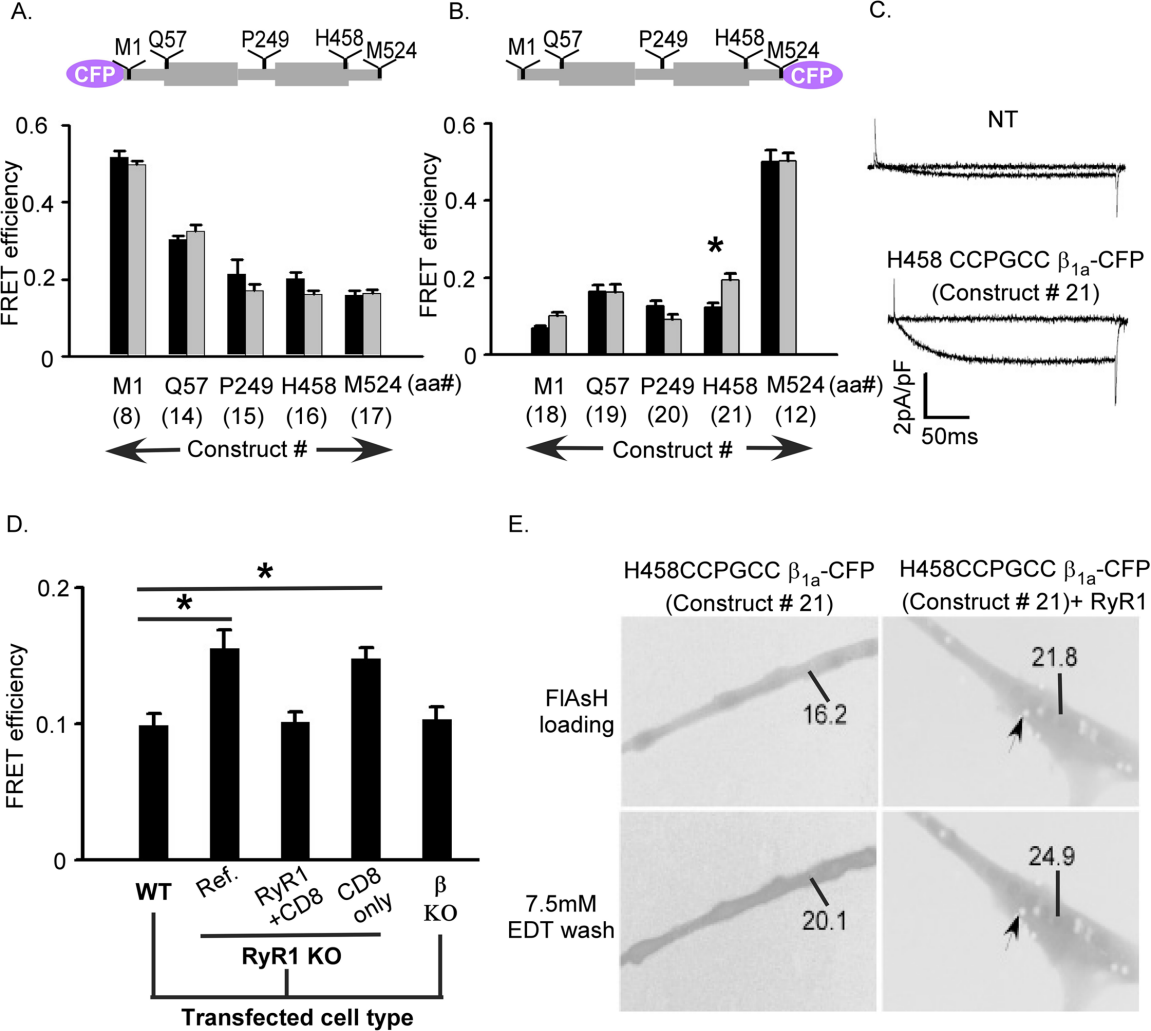
Intramolecular FRET in the ß_1a_ subunit depends upon donor-acceptor position and RyR1 expression. (A) FRET efficiencies for ß_**1a**_ subunit constructs in myotubes when CFP donor was located at the N-terminus and CCPGCC acceptor site was tested at five different positions of ß_**1a**_ labeled M1 (construct 8), Q57 (construct 14), P249 (construct 15), H458 (construct 16) and M524 (construct 17), as indicated in schematic above bar graphs. (B) FRET efficiencies for same ß_**1a**_ subunit constructs in myotubes except the CFP donor was located at the C-terminus (construct 12, 18, 19, 20 and 21). For both panels A and B the ß_**1a**_ constructs were expressed in wild type (black) or RyR1 KO (gray) myotubes. Only the construct H458-CCGPCC ß_**1a**_-CFP (construct 21) displayed significant difference FRET efficiency (*p<0.001) in the presence (in WT 0.12 ± 0.01) versus absence (in KO 0.19 ± 0.01) of RyR1 (in Table 1 boxed). (C) Ca^**2+**^ current expression at test potentials of –30 mV and +30 mV in ß_**1**_ KO myotubes expressing H458-CCGPCC ß_**1a**_-CFP (construct 21) compared to non-transfected (NT) ß KO control which demonstrates that this construct forms functional ß subunits. (D) Comparison of FRET efficiency of H458 CCPGCC ß_**1a**_-CFP (construct 21) transfected in either WT or dyspedic myotubes. Transfection of RyR1 into dyspedic myotubes eliminated the increase in FRET observed in the absence of RyR1. The RyR1 expression marker (CD8) or the presence of endogenous ß_**1a**_ subunits has no effect on the measured FRET efficiencies. The FRET efficiency of construct 21 was 0.12 ± 0.01, when it was transfected in either wild type (WT) or ß KO or RyR1 KO cells in presence of RyR1 + CD8 plasmids (triple transfected). Additionally a comparable FRET efficiency observed in RyR1 KO cells between construct 21 alone (0.19 ± 0.01) and construct 21 with CD8 plasmid co-transfection (0.18 ± 0.01), which was significantly higher than the WT (*p<0.001; see results in Table 1) (E) Expression and intramolecular FRET of H458CCPGCC ß_**1a**_- CFP alone and co-transfected with RyR1 in dyspedic myotubes with the number represents the fluorescence intensity of the region in the myotube and the arrow indicated CD8 beads.

To map the domains of ß_1a_ by FRET we kept the donor (CFP) position fixed at either the N-terminus or C-terminus and introduced the acceptor (CCPGCC motif) at the same five critical positions described above in ß_1a_ molecule (constructs 8, 12 and 14 to 21). FRET efficiency measurements were conducted in wild type as well as dyspedic (lacking RyR1) myotubes. In general, comparatively weaker FRET was detected when CFP and the tetracysteine tag were separated by intervening ß_1a_ sequence compared to inserted CFP-CCPGCC concatemers; however, for each of the inserted positions high FRET efficiency was observed (Fig 4A and 4B). The results were indistinguishable between wild type and dyspedic myotubes for all constructs except for a significant difference (p<0.001) in case of H458 CCPGCC ß_1a_-CFP (construct 21). The FRET efficiency at this location was 0.12 ± 0.01 in wild type myotubes and 0.19 ± 0.01 in dyspedic myotubes (Fig 4B, construct 21 and Table 1). These data indicate a conformational change in the C-terminus of ß_1a_ induced by the presence of RyR1. They lower FRET between excitation pairs at H458 CCPGCC and the C-terminus CFP further suggests that the C-terminal region assumes a more extended conformation in the presence of RyR1.

**Table 1:** FRET efficiency of N-terminal or C-terminal CFP and CCPGCC tagged in different domain of ß_1a_ constructs expressed in WT, dyspedic and ß_1_ KO myotubes.

cDNA Construct	Transfected Cell Type	No. of cells analyzed	FRET efficiency
**CFP-ß**_**1a**_			
Q57 CCPGCC	WT	40	0.30±0.01
(Construct # 14)	RyR1 KO	41	0.32±0.01
P249 CCPGCC	WT	19	0.21±0.03
(Construct # 15)	RyR1 KO	19	0.17±0.01
H458 CCPGCC	WT	35	0.20±0.01
(Construct # 16)	RyR1 KO	23	0.16±0.01
M524 CCPGCC	WT	46	0.15±0.01
**ß**_**1a**_ **-CFP**			
M1 CCPGCC	WT	44	0.06±0.01
(Construct # 18)	RyR1 KO	43	0.01±0.01
Q57 CCPGCC	WT	26	0.16±0.01
(Construct # 19)	RyR1 KO	20	0.16±0.02
P249 CCPGCC	WT	33	0.12±0.01
(Construct # 20)	RyR1 KO	30	0.09±0.01
H458 CCPGCC	WT	37	0.12±0.01
(Construct # 21)	RyR1 KO	23	0.19±0.01
H458 CCPGCC	RyR1 KO	34	0.12±0.01
(Construct # 21) + RyR1 + CD8			
H458 CCPGCC	RyR1 KO	31	0.18±0.01
(Construct # 21) + CD8			
H458 CCPGCC	ß_1_ KO	35	0.12±0.01
(Construct # 21)			

The H458 CCPGCC ß_1a_-CFP construct (construct 21) was then subjected to additional analysis given the difference in FRET observed between wild type and dyspedic myotubes. First, we demonstrate that this construct can form functional ß subunits based on its ability to rescue L-type Ca^2+^ currents in ß_1_ KO myotubes (Fig 4C). To determine if the observed difference between wild type and dyspedic myotubes for the construct H458 CCPGCC- ß_1a_-CFP (construct 21) was specifically due to the absence of RyR1, dyspedic myotubes were transfected with H458 CCPGCC- ß_1a_-CFP (construct 21), wild type RyR1 and CD8 (CD8 to allow detection of the transfected myotubes). The RyR1 + CD8 dyspedic myotubes showed FRET (0.12 ± 0.01) comparable to wild type myotubes (Fig 4D and 4E right panel and Table 1), and expression of CD8 alone in dyspedic myotubes resulted in FRET (0.18 ± 0.01) indistinguishable from nontransfected dyspedic myotubes (Fig 4D). These results support the idea that the RyR1 is the critical difference inducing a conformational change in the ß_1a_ subunit C-terminus. An additional control experiment measured the FRET efficiency of the construct H458 CCPGCC ß_1a_-CFP (construct 1) in ß_1_ KO myotubes to exclude an effect of native ß_1_ subunits, and the measured FRET efficiency was similar (0.12 ± 0.01) to that observed in wild type myotubes (0.12 ± 0.01). Overall, these experiments indicate that the C-terminus of the ß_1a_ subunit exhibits significantly different conformations in the presence and the absence of RyR1 in resting myotubes.

## Discussion

In the present study, we have demonstrated a conformational change of ß_1a_ induced by RyR1 in native skeletal myotubes using a FlAsH-based *ex vivo* FRET method. The FlAsH-based *ex vivo* FRET assay can report proximity relationships for donor/acceptor pairs in skeletal myotubes under appropriate experimental (native) conditions. This method also eliminates potential limitations for the use of FlAsH as a FRET acceptor (non-specific binding and limited access to tetracysteine binding sites) for intramolecular FRET. In this study, a robust intramolecular FRET of the DHPR ß_1a_ subunit using 10 different donor/acceptor pairs spanning all domains of the protein demonstrated the relatively compact nature of the protein. Furthermore, the conformation of the DHPR ß_1a_ subunit was specifically altered in the C-terminus by the presence of the RyR1 receptor suggesting an interaction between these two proteins.

### Implications for in situ structure of DHPR ß_1a_ subunit

**Fig 5:**
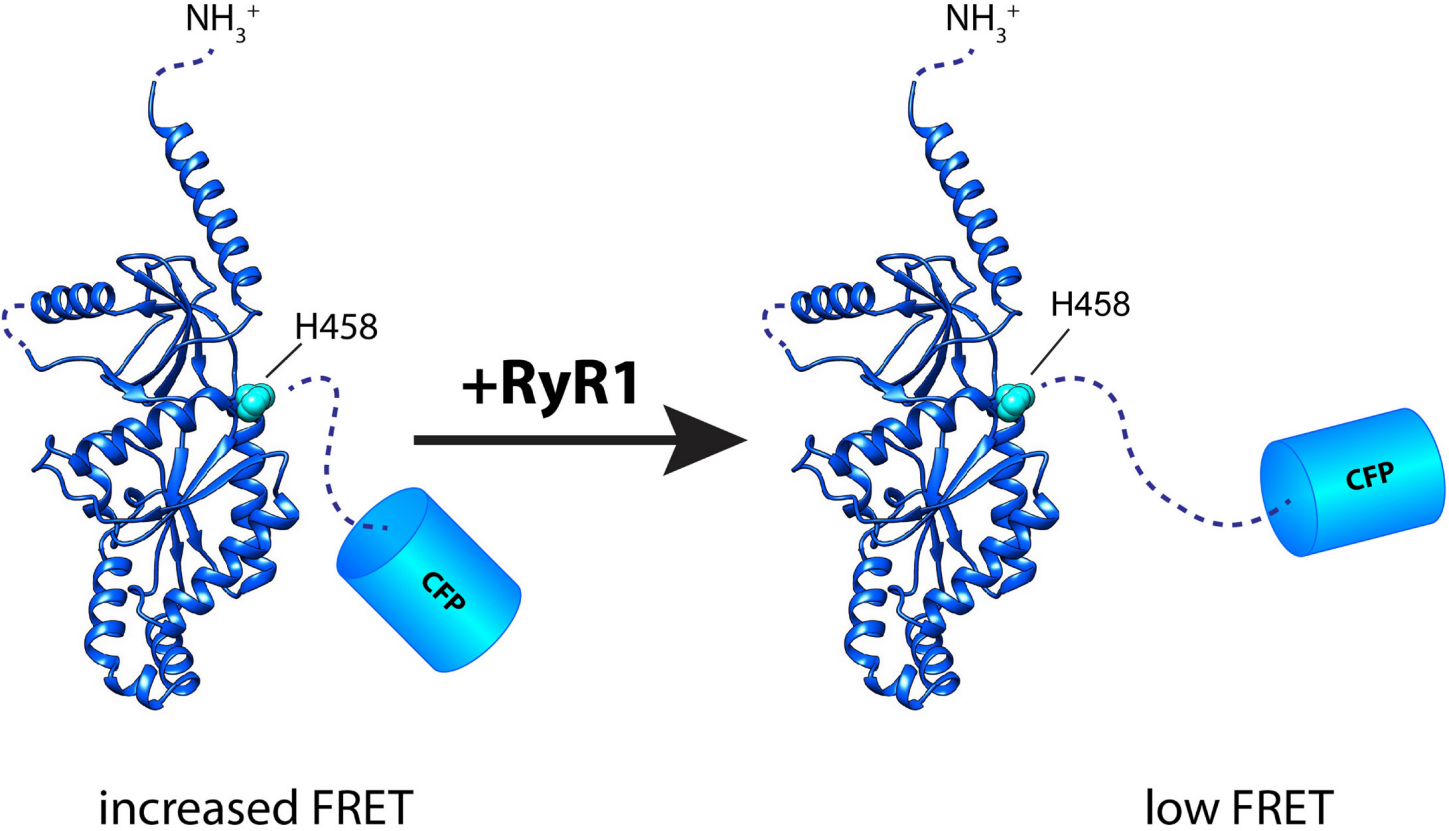
Model of H458 CCPGCC ß_1a_- CFP in a FRET competent state. The ß_**1a**_ subunit structure was predicted with Phyre2 and obtained using ß_**3**_ subunit as a template. The lower FRET between excitation pairs at H458 (CCGPCC) and the C-terminus (CFP) in the presence of RyR1 suggests a structural change wherein the C-terminal region of the modeled ß_**1a**_ assumes an extended conformation.

X-ray crystallographic studies defined the structure of the central core of ß_2_, ß_3_ and ß_4_ subunits revealing interlinked SH3 and GK domains representative of members of the MAGUK superfamily of adapter proteins [29–31]. A less ordered domain in between the SH3 and GK domains composed of the variable central domain of ß subunits is comparable to the ‘hook’ domain described for the related MAGUK protein PSD95, which may be important for interacting with other proteins [51]. The organization of the SH3 and GK domains in the ß subunits takes a more elongated conformation compared to the more compact relationship of these domains in PSD95[31]. Neither the N- nor C-terminal sequences were resolved in these structures and are likely disordered. Despite the fact that the ß_1_ subunit structure has not been solved, extensive sequence homology between all ß subunit core regions (> 60% identity between ß_1_ and the other subunits) suggests a comparable structural organization. This identity was exploited to model the structure of the ß_1a_ subunit (Fig 5). The model maintains the core domains and properly positions conserved residues at the binding site for the œ interaction domain (AID). Notably, this model also suggests that H458, a CCPGCC insertion site, is the last residue preceding the flexible C-terminus. Thus, the present intramolecular *ex vivo* FRET data for the ß_1a_ subunit can reasonably be compared to the solved crystallographic structures for the ß subunits.

In general, FRET measurements and X-ray crystallography data can provide complementary information on the structural arrangement of a protein. Although the spatial resolution and overall structural detail is less for FRET measurements compared to X-ray diffraction, the unique advantages of FRET include the ability to make measurements in living cells and to detect changes in conformation in response to acute interventions. Because FRET is dependent on both the distance separating the donor and acceptor as well as their relative orientation, it is not possible to calculate absolute changes in distances between donor and acceptor without verifying the orientations [52]. Nevertheless, given the steep dependence (sixth power) of FRET on the distance separating donor and acceptor, it is possible to provide upper limit estimates of distance separating regions in that FRET will not be observed at separations of more than ~10 nm [53]. Because CFP on both the amino and carboxyl termini was able to exhibit FRET from FlAsH-mediated binding to tetracysteine motifs spanning all domains of the ß_1a_ subunit, we conclude that the overall structure of the ß_1a_ subunit is relatively compact with a diameter of less than 10 nm from either the carboxyl or amino terminus. While this is consistent with published structures and our model, this overall architecture cannot be presumed from the crystal structures alone as the native N- and C-termini are absent. Thus, the major new structural information is that *in situ* neither the amino or carboxyl termini are in an extended conformation, but instead they fold over or near the central core region of the ß_1a_ subunit.

DHPR ß subunits have previously been shown to exhibit trans-complementation of the SH3 and GK domains suggesting the formation of dimeric or higher order multimeric complexes of ß subunits [48–50]. In addition, other MAGUK proteins can exhibit intermolecular binding of SH3 and GK domains [54–56], and this type of three-dimensional domain swapping has been described for a variety of proteins [57]. In the case of MAGUKs, multimerization has been proposed to be regulated by factors acting on the HOOK domain between the SH3 and GK domains [54, 55]. However, typically the intramolecular assembly of SH3-GK domains in MAGUKs predominates and effectively competes with the intermolecular SH3-GK interactions [56, 58–60]. For the ß subunits, the biology is intriguing in that not only are full-length ß subunits described, but also truncated ß subunits, which lack the GK domain and carboxyl terminus due to alternative splicing, have been described in native tissues [61–63]. Thus there are multiple possibilities for intermolecular interactions between ß subunits expressed in native cells where a variety of different ß subunit isoforms may be present. However, the current experiments in myotubes did not detect any evidence for intermolecular interactions between full-length ß_1a_ subunits with amino terminus tags. This finding is consistent with the observed stoichiometry of 1:1 for purified DHPR œ_1S_ and ß subunits from skeletal muscle [64]. Nevertheless, regulated intermolecular assembly of different DHPR ß subunit isoforms will likely be an important focus of future study.

### Role of ß_1a_ in skeletal excitation-contraction coupling

The core of DHPR ß subunits which includes only the SH3 linked to GK domain has been found to be adequate to recapitulate fundamental functional effects including membrane trafficking of the channel complex as well as modulating voltage-dependent gating [48–50]; however, the full-length ß_1a_ subunit including the carboxyl terminus is required for skeletal type EC coupling in mouse myotubes [28]. Studies in the zebrafish relaxed mutant, which lacks ß_1a_ protein expression, revealed an essential role of ß_1a_ in the formation of tetrads (groups of four DHPRs) and thus physical DHPR-RyR1 interactions [65]. A unique heptad repeat in the C-terminus of ß_1a_ has been identified as essential for skeletal EC coupling in functional studies [28]. Therefore, a physical contact between C-terminus of the DHPR ß_1a_ and RyR1 has been hypothesized, and consistent with this hypothesis, the C-terminal 35 residues of ß_1a_ with an essential hydrophobic triplet (L^496^, L^500^ and W^503^) increase RyR1 channel activity in lipid bilayers [14, 66]. Biochemical pull-down techniques also identified a region of positively charged amino acids in the RyR1 (3495–3502) that specifically interact with the DHPR ß_1a_ [13]. The present study supports a critical functional interaction, in native conditions, between the C-terminus of ß_1a_ and RyR1. FRET between the H458 CCPGCC insertion and the C-terminal CFP significantly increased in RyR1 knockout myotubes (Fig 4B and 4D). This suggests that RyR1 induces a conformational change within ß_1a_ where its C-terminus assumes a more extended structure (i.e. lower FRET state) (Fig 5). This conformational plasticity may be critical between ß_1a_ and RyR1 activities in cytoplasm, and the high degree of sequence diversity within the disordered C-terminus of the different ß subunits may contribute to the specificity of this interaction. However, a previous study using a tandem CFP-YFP reporter inserted at the amino or carboxyl terminal of ß_1a_ revealed a significant change in FRET in myotubes induced by RyR1 expression for the amino terminal tagged construct and not the carboxyl terminal tagged protein, and this contrasts the present results in which there were no differences in FRET for CFP-tetracysteine concatemers expressed at the amino or carboxyl termini [38]. The larger size of the dual fluorescent protein reporter system relative to the CFP-tetracysteine concatemer used in the present *ex vivo* FRET study may contribute to these different results in which general spatial constraints may be apparent only with the larger protein reporter system [36]. Although it was suggested that the amino terminus is more proximal to the RyR1 than the carboxyl terminus, alternative explanations exist in which the overall three dimensional protein complex organization can be altered by RyR1 expression which results in changes in spatial restraints due to a number of associated proteins, e.g. œ_1s_, rather than just proximity to RyR1. A recent study using FRET techniques has indicated that association of I-II loop of DHPR œ(1S) subunit with RyR1 causes reorientation of multiple cytoplasmic domains of the dihydropyridine receptor [8].

A model of skeletal muscle EC coupling is emerging which involves multiple physical contacts between the DHPR complex and RyR1, and one of the essential physical links occurs via the DHPR ß_1a_ subunit. How such links can transmit the voltage-dependent gating conformational changes occurring in the sarcolemmal DHPR œ_1s_ to the RyR1 in the sarcoplasmic reticulum is an important area of future study. Interestingly, structural studies have suggested that ß subunit binding to the AID in the I-II linker of œ subunits can cause a coil to helix transition, which could result in a continuous œ-helix from IS6 through the AID thus providing a relatively rigid physical link between the voltage sensing α subunit and the ß subunit [30, 31]. The present study provides evidence for an interaction between the ß_1a_ subunit and RyR1 in resting myotubyes, but future studies are needed to refine this working model of skeletal EC coupling which may include dynamic intramolecular ß_1a_ subunit FRET measurements during channel gating and determination of intermolecular FRET between DHPR subunits and RyR1.
